# Telocytes: novel interstitial cells present in the testis parenchyma of the Chinese soft‐shelled turtle *Pelodiscus sinensis*

**DOI:** 10.1111/jcmm.12731

**Published:** 2015-11-27

**Authors:** Ping Yang, Nisar Ahmad, Yufei Hunag, Shakeeb Ullah, Qian Zhang, Yasir Waqas, Yi Liu, Quanfu Li, Lisi Hu, Qiusheng Chen

**Affiliations:** ^1^ Key Laboratory of Animal Physiology and BiochemistryMinistry of AgricultureCollege of Veterinary MedicineNanjing Agricultural UniversityNanjingChina

**Keywords:** telocytes, testes, Chinese soft‐shelled turtle (*Pelodiscus sinensis*), ultrastructure, CD34

## Abstract

Telocytes (TCs) are novel interstitial cells that have been found in various organs, but the existence of TCs in the testes has not yet been reported. The present ultrastructural and immunohistochemical study revealed the existence of TCs and differentiate these cells from the peritubular cells (Pc) in contact with the surrounding structures in the testes. Firstly, our results confirmed the existence of two cell types surrounding seminiferous tubules; these were Pc (smooth muscle like characteristics) and TCs (as an outer layer around Pc). Telocytes and their long thin prolongations called telopodes (Tps) were detected as alternations of thin segments (podomers) and thick bead‐like portions (podoms), the latter of which accommodate the mitochondria and vesicles. The spindle and irregularly shaped cell bodies were observed with small amounts of cytoplasm around them. In contrast, the processes of Pc contained abundant actin filaments with focal densities, irregular spine‐like outgrowths and nuclei that exhibited irregularities similar to those of smooth muscle cells. The TCs connected with each other *via* homocellular and heterocellular junctions with Pc, Leydig cells and blood vessels. The Tps of the vascular TCs had bands and shed more vesicles than the other TCs. Immunohistochemistry (CD34) revealed strong positive expression within the TC cell bodies and Tps. Our data confirmed the existence and the contact of TCs with their surroundings in the testes of the Chinese soft‐shelled turtle *Pelodiscus sinensis*, which may offer new insights for understanding the function of the testes and preventing and treating testicular disorders.

## Introduction

Testes are components of both the reproductive and endocrine systems. The main functions of the testis are the production of spermatozoa (spermatogenesis) and androgens, primarily testosterone 1. The testis parenchyma consists of seminiferous tubules and interstitial tissue. The testis interstitial tissue (TIS) is highly vascularized loose connective tissue that includes Leydig cells, blood vessels, leukocytes and fibroblasts. Spermatogenesis occurs in the seminiferous tubules, which are composed of Sertoli cells (SCs) and maturing germ cells that are surrounded by one (for example in rats and mice) or more (*e.g*., humans and turtles) layers of peritubular cells (Pc). In either case, the Pc are elongated, very thin, and spindle‐shaped and express the characteristics of smooth‐muscle‐like cells because they contain abundant actin filaments and other cytoskeletal proteins 2, 3. Hence, according to the description of Pc, one outer layer of cells around the Pc has confused the description of a novel cell type known as telocytes (TCs). Therefore, we hypothesized that the some Pc (in the outer layer) are in fact TCs.

Telocytes are a special type of interstitial cells that was recently identified by Popescu's research group 4, 5, 6, and the concept of TCs has been adopted by many laboratories and organs worldwide 4, 7, 8, 9, 10, 11, 12, 13, 14, 15, 16, 17, 18, 19, 20, 21, 22. In 2005, a new cell type was described and termed interstitial Cajal‐like cells (ICLCs) based on their morphological similarities to gastrointestinal interstitial Cajal cells (ICC). Interstitial Cajal‐like cells were recently distinguished and separated from ICC and/or other interstitial cells based on their special features. Finally, Popescu renamed ICLCs as TCs in 2010 based on their extremely long prolongations 4. Telocytes are ultrastructurally characterized by a small cell body and very long processes that are called telopodes (Tps) and display moniliform aspects involving alternating thin segments (podomers) and dilated regions (podoms) 4, 23, 24, 25. Telopodes can range in length from several tens to hundreds of micrometres and form junctions with a number of cells 3, 4, 11, 26, 27. Telocytes release ectosomes/exosomes usually as cargos 11, 12, 26. These cells are not excitable 13, 28. At present, transmission electron microscopy (TEM) is the best available method for the accurate identification of TCs. Recent studies have described the gene expression profiles, microRNA signatures, proteomic profiles and electrophysiological characteristics of TCs 29, 30, 31, 32. Several roles have been suggested for TCs. They have been proposed to play a mechanical support role that ensures the correct organization of the connective tissues within organs 23, 33. Telocytes may be involved in intercellular signalling between stromal cells, smooth muscle cells, microvessels, immune cells and nerve bundles *via* the paracrine secretion of signalling molecules and cell‐to‐cell contacts 3, 8, 15, 34, 35. These cells may also act in neurotransmission by spreading the slow waves generated by the pacemaker ICCs 23, 36. Telocytes with similar ultrastructural and phenotypic characteristics have been found in many organs 3, 4, 5, 6, 7, 8, 9, 10, 11, 12, 13, 14, 15, 16, 17, 18, 19, 20, 21, 22, 23, 24, 25, 26, 28, 29, 37. These findings have led to the assumption that TCs may exist in all organs. However, it remains unknown whether TCs are present in the testes. To the best of our knowledge, this study provides the first demonstration that TCs exist in the testes in a form that differentiates them from Pc.

## Materials and methods

### Animals

Five male adult soft‐shelled turtles, *Pelodiscus sinensis* were purchased from a wild breeding base in Jiangsu province of China. Turtles, weighing 0.8–1 kg each, were housed in a local facility for laboratory animal care and held, fed *adlibitum*, according to the local ethical guidelines. All animals were rendered comatose using sodium pentobarbital (1 ml/animal) administered intraperitoneally and were killed at one time by cervical dislocation. The study was performed according to accepted international standards and approved by the Ethic Committee for Animal Care and Use, by Science and Technology Agency of Jiangsu Province (SYXK (SU) 2010‐0009).

### Light microscopy

Specimen were placed in 10% neutral buffered formalin for fixation, and then embedded in paraffin wax and wax blocks were prepared. Sectioning was done at 5 μm. These sections were stained with haematoxylin and eosin staining for light microscopic analysis using Olympus microscope (BX53, Tokyo, Japan), camera (Olympus DP73).

### TEM

The specimen were cut into small parts and then immersed in 2.5% glutaraldehyde in PBS (4°C, pH 7.4, 0.1 M) for overnight. Tissue were rinsed in the same PBS and then post‐fixed for 60 min. at room temperature by using buffered 1% osmium tetroxide (Polysciences Inc., Warrington, PA, USA) and washed in the buffer. The samples were then dehydrated in ascending concentrations of ethyl alcohol, infiltrated with a propylene oxide–Araldite mixture and then embedded in Araldite. The blocks were then sectioned by using an ultramicrotome (ReichertJung, Wien, Austria) and the ultrathin sections (50 nm) were mounted on cooper coated grids. The pieces were stained with 1% uranyl acetate and Reynold's lead citrate for 20 min. Finally samples were examined and photographed by using a high resolution digital camera (16 mega pixel) connected to the TEM, Hitachi H‐7650 (Toyko, Japan).

### IHC

Paraffin sections (6 μm) were placed on poly‐l‐lysine‐treated glass slides and were stained according to immunohistochemical standard techniques. Briefly, after deparaffinization and washing in PBS, these sections were covered with 3% hydrogen peroxide in PBS for 15 min. at 37°C to block the further activity of endogenous peroxidase. The samples were blocked with 5% bovine serum albumin and incubated with rabbit anti CD34 (1:100) antibody (Santa Cruz Biotechnology, Dallas, TX, USA) in a moisture chamber at 40°C for 24 hrs. After washing, the sections were incubated with biotinylated anti‐rabbit IgG (Boster Bio‐Technology, Wuhan, China) for 1 hour at room temperature. The sections were then rehydrated in PBS (pH 7.2), incubated with avidin‐biotinylated peroxidase complex for 45 min. at 37°C. After being washed with PBS, peroxidase activity was revealed using DAB (Boster Bio‐Technology, Wuhan, China) according to the instructions of company.

## Results

**Figure 1:**
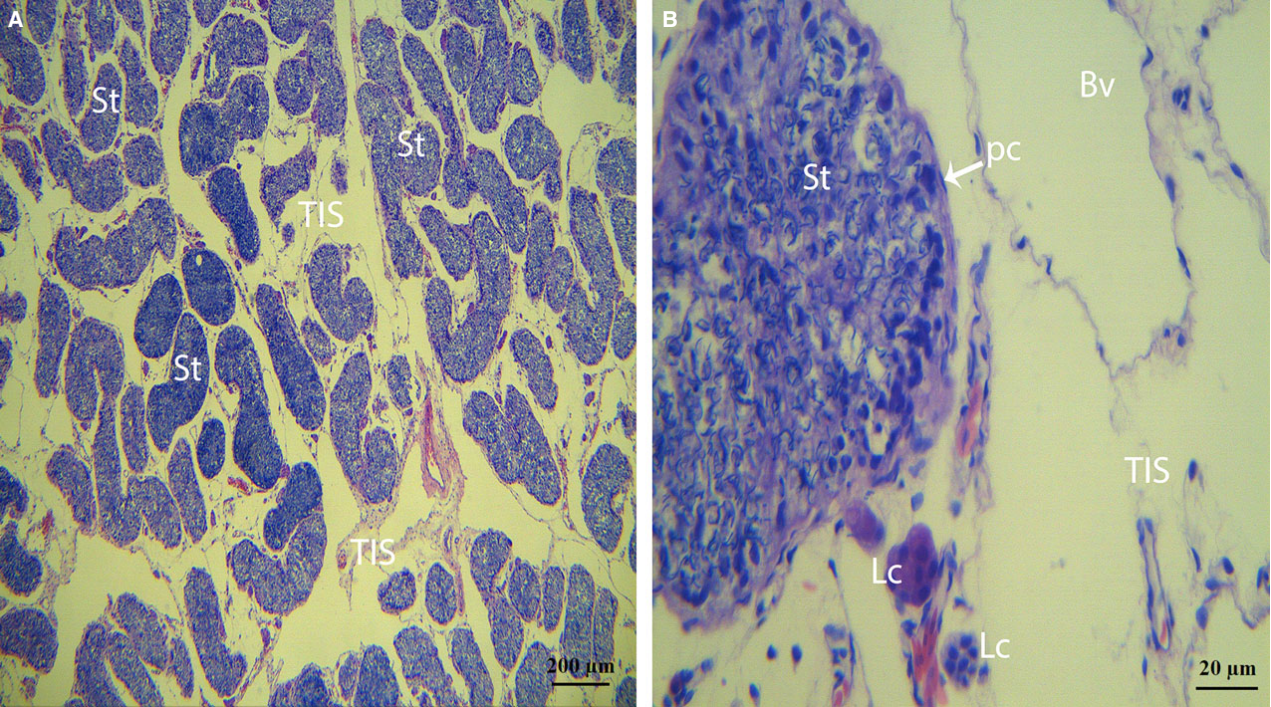
Photomicrograph of a turtle testis for haematoxylin and eosin staining. (**A**) The parenchyma exhibits many seminiferous tubules and testis interstitial tissue. (**B**) Peritubular cells are present within the walls of the seminiferous tubules, and Leydig cells are clearly observed within the interstitial tissue. The walls of the blood vessels consist of endothelial cells with clear lumina and small amounts of fibres from the other side (St: seminiferous tubules; Bv: blood vessel; Pc: peritubular cells; Lc: Leydig cell; TIS: testis interstitial tissue). The scale bars represent 200 μm (**A**) and 20 μm (**B**).

Light microscopy revealed many seminiferous tubules within the parenchyma of the turtle testes (Fig. 1A). In the walls of the seminiferous tubules, several layers of Pc were observed. The TIS lies between the seminiferous tubules, which are formed of highly loose connective tissue. The TIS has been observed to display very distinct characteristics, such as abundant vessels (blood vessels and lymphatic vessels), thin fibres and a small number of cells. Leydig cells (*i.e*., the interstitial cells of Leydig) were found adjacent to the seminiferous tubules within the interstitial tissue (Fig. 1A and B).

### TEM

**Figure 2:**
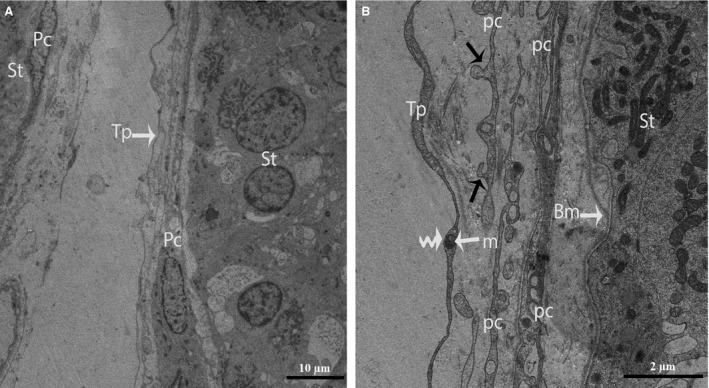
TEM micrograph of a turtle testis. (**A**) Several layers of peritubular cells are present within the walls of the seminiferous tubules, and long Tps border the peritubular cells. (**B**) The processes of the peritubular cells exhibit short, thick, unidirectional outgrowths (black arrow) and are located near the basal membrane of the seminiferous tubule. The peritubular cells also shed vesicles (arrow head). The mitochondria are clearly visible in the podom (curved arrow) of the Tp. Pc: peritubular cell; Tp: telopode; Bm: basal membrane; St: seminiferous tubules. The scale bars represent 10 μm (**A**) and 2 μm (**B**).

**Figure 3:**
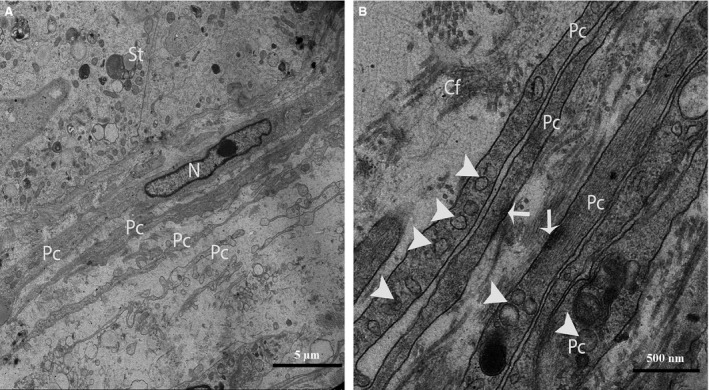
TEM micrograph of a turtle testis. (**A**) Several layers of peritubular cells are present near the seminiferous tubules, and the nuclei exhibit many irregularities (concertina nuclei). (**B**) The processes of the peritubular cells exhibit abundant actin filaments as well as dense focal bodies (arrows) and caveolae (arrowheads). St: seminiferous tubules; Pc: peritubular cell; N: Nucleus; Cf: collagen fibre. The scale bars represent 5 μm (**A**) and 500 nm (**B**).

Through TEM, several layers of Pc were clearly observed in the walls of the seminiferous tubules (Fig. 2A and B). Some Pc presented the typical characteristics of smooth muscle cells, such as nuclei that exhibited many irregularities (*i.e*., concertina nuclei) (Fig. 3A), abundant actin filaments with dense focal bodies, and caveolae (Fig. 3B). The Pc located in the inner layer (near the seminiferous tubules) exhibited thicker processes and contained more actin filaments and dense material than the cells located in the outer layer (Fig. 3B).

**Figure 4:**
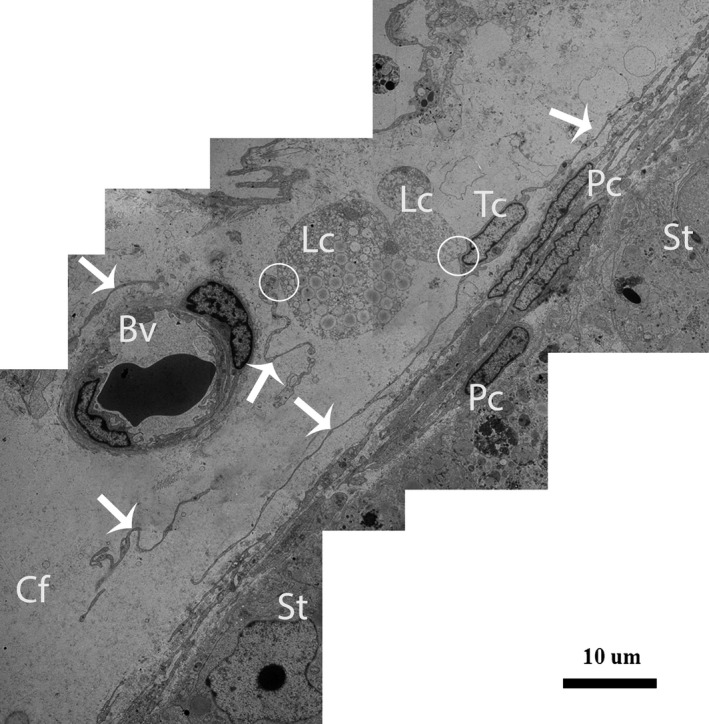
TEM micrograph of a turtle testis. A TC with very long thin prolongations (Tps, arrows) bordering several layers of peritubular cells. The encircled area indicates the heterocellular junction (point contact) between TCs and Leydig cells. Several Tps are present in close proximity to blood vessels and Leydig cells within the interstitial tissue. St: seminiferous tubules; Tc: telocyte; Pc: peritubular cell; Lc: Leydig cell; Cf: collagen fibre, Bv: blood vessels. The scale bar represents 10 μm.

**Figure 5:**
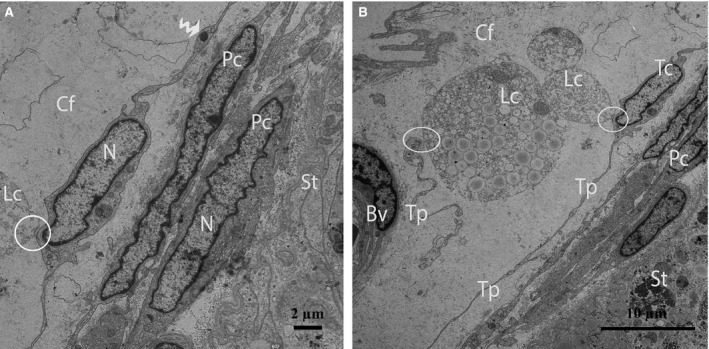
TEM micrograph of a turtle testis (close view of Fig. 4). (**A**) Peritubular cells exhibiting the typical concertina‐like nuclei are located closest to the seminiferous tubules. An image of a TC with a spindle‐shaped cell body containing Tps and a small amount of cytoplasm surrounding the nucleus is shown. The curved arrow indicates a podom in which mitochondria are clearly visible. (**B**) The encircled area displays the close contact between TCs and Leydig cells. The Tps exist near peritubular cells and a blood vessel. St: seminiferous tubules; Pc: peritubular cell; Tc: telocyte; Tp: telopode; m: mitochondria; Lc: Leydig cell; Bv: blood vessel. The scale bars represent 2 μm (**A**) and 10 μm (**B**).

**Figure 6:**
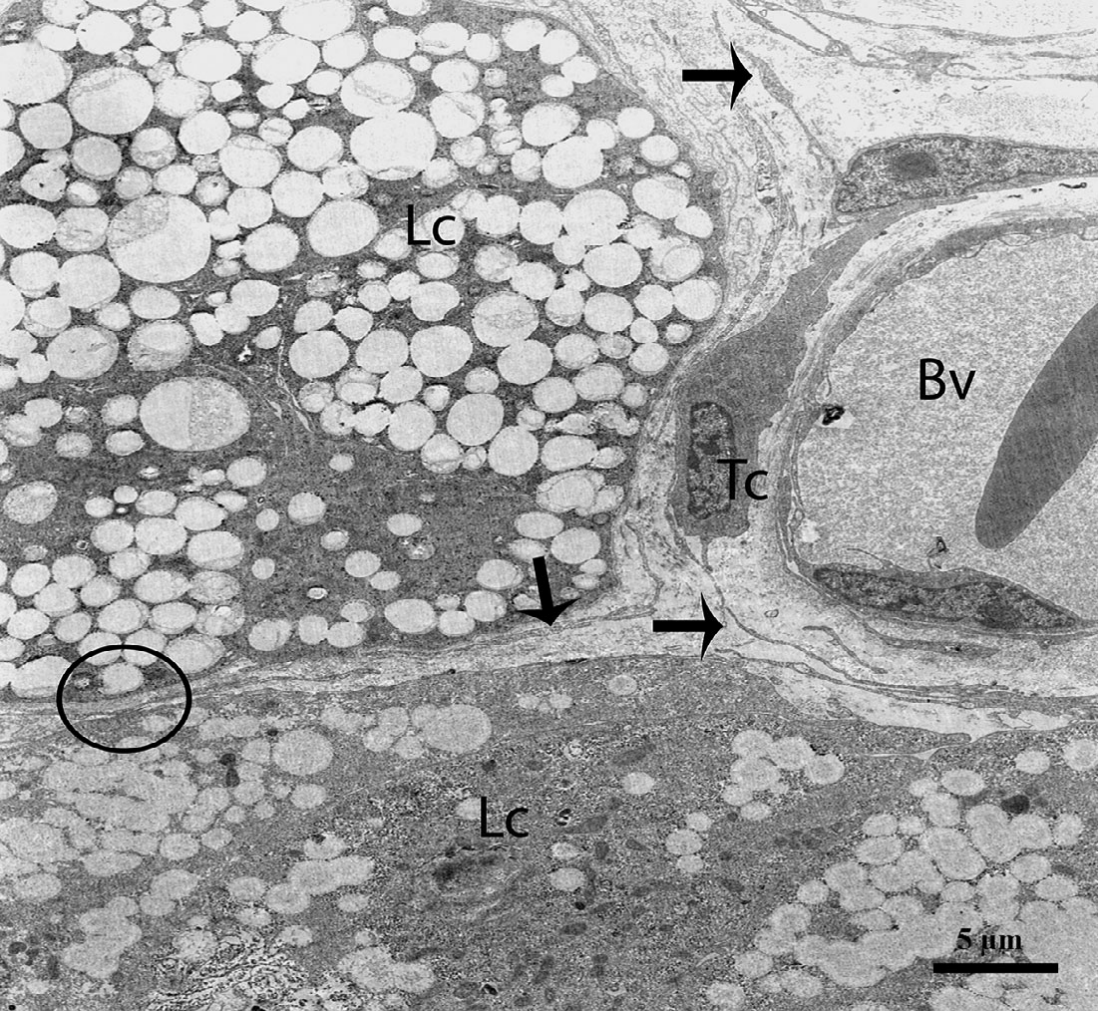
TEM micrograph of a turtle testis. A typical telocyte and several Tps (arrows) are present near to Leydig cells and a blood vessel. The encircled area shows the heterocellular junction between a Tp and a Leydig cell. Tc: telocyte; Lc: Leydig cell; Bv: blood vessel. The scale bar represents 5 μm.

After carefully observing the outermost layers of the Pc, a novel type of interstitial cell was often observed (Figs. 2A, B and 4). These cells were identified as TCs according to the diagnostic criteria for TCs suggested by Popescu and Faussone‐Pellegrini 4. However, in terms of morphology, these cells are easily confused with Pc because both cells types have long processes. Figure 4 shows a typical TC with two extremely long processes (Tps) near the Pc. The observation of the TCs revealed that the cell body was small, the nuclei exhibited moderate heterochromatin at the periphery with a small amount of cytoplasm surrounding the nuclei, and the cytoplasm contained numerous mitochondria. In addition, the prolongations extended from the cell bodies and were more than one hundred micrometres in length. Furthermore, the observed Tps (cellular extensions) exhibited alternating thin segments (podomers) and dilated bead‐like regions (podoms). The podoms accommodated the mitochondria and vesicles (Figs. 2B and 5A). Whereas the processes of the Pc were thicker, and greater numbers of spine‐like short process were found (Figs. 2B and 3A, B). Additionally, the appearances of the nuclei of the Pc were similar to those of smooth muscle cells (*i.e*., concertina nuclei) (Figs. 3A and 5A). Several Tps were scattered in the interstitial tissue along with few fibres around the blood vessels and Leydig cells. Close membrane‐to‐membrane contacts between the TCs and Leydig cells were observed (Figs. 4 and 5A, B). The TCs shared obvious substantial connections with the Leydig cells. As shown in Figure 6, the TCs presented close contacts with Leydig cells on one side and a large blood vessel on the other side. Numerous Tps bordered the Leydig cells and formed closed membrane‐to‐membrane heterocellular junctions, whereas the Leydig cells were found in clusters of variable size. The cytoplasm was strongly granular with numerous lipid‐filled vesicles, and the nuclei were large and round (as suggested by Naraghi) 38 (Fig. 6).

**Figure 7:**
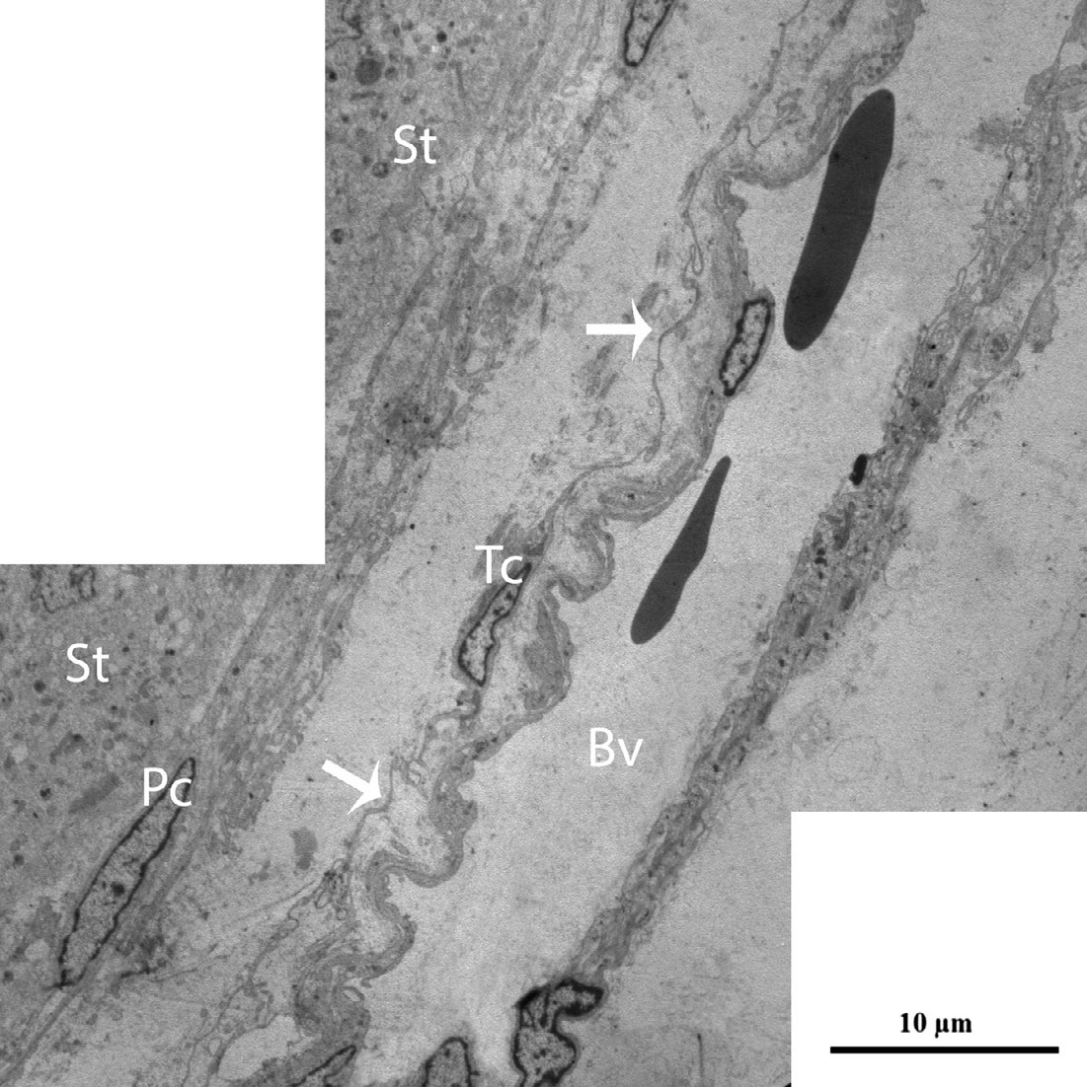
TEM micrograph of a turtle testis. TCs with extremely long processes (arrow) bordering a large blood vessel exist between peritubular cells and a blood vessel within the interstitial tissue. St: seminiferous tubules; Pc: peritubular cell; Tc: telocyte; Tp: telopode; Bv: blood vessels. The scale bar represents 10 μm.

**Figure 8:**
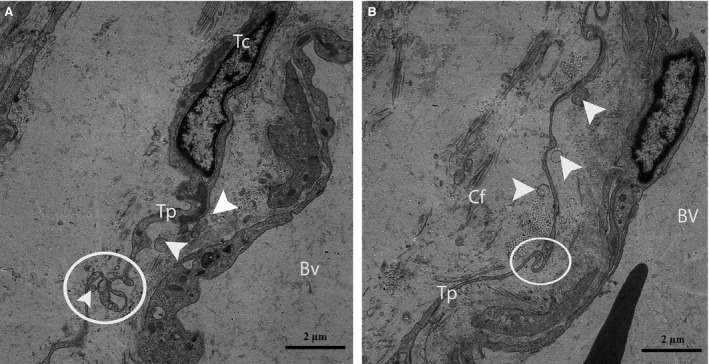
TEM micrograph of a turtle testis (magnified view of Fig. 7). A typical Tp of a telocyte displaying the band (circular area) around a blood vessel located within the interstitial tissue is shown. The Tp also shed different types of vesicles (arrowheads) near the blood vessels (**A** and **B**). Tc: telocyte; Tp: telopode; Bv: blood vessel; Cf: collagen fibre. The scale bar represents 2 μm (**A** and **B**).

**Figure 9:**
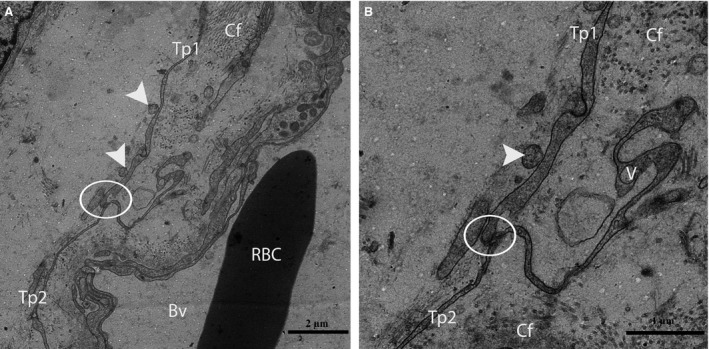
TEM micrograph of a turtle testis. (**A**) The circular area shows the nanocontact (end‐to‐side) between two Tps around a large blood vessel located within the interstitial tissue. Vesicles (arrowheads) are clearly visible around the Tp. (**B**) A higher magnification view of **A** shows the nanocontact between two Tps and vesicles. St: seminiferous tubules; Tp: telopode; Bv: blood vessels; Cf: collagen fibre; RBC: red blood cell. The scale bars represent 2 μm (**A**) and 1 μm (**B**).

**Figure 10:**
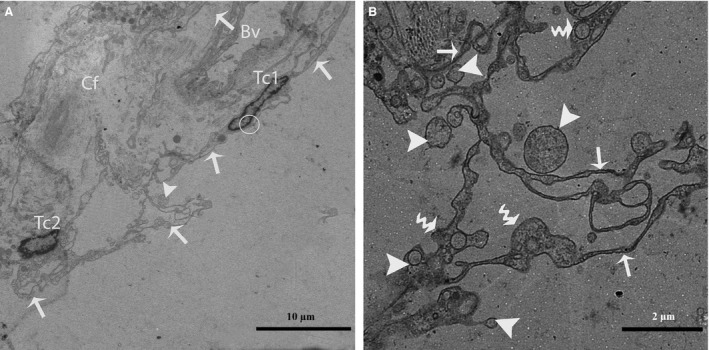
TEM micrograph of a turtle testis. (**A**) Two TCs exhibiting several Tps (arrow) that arise directly from the cell bodies and further divide into several branches in a dichotomous pattern are shown. Tps are present in close proximity to a blood vessel, and a small number of collagen fibres are also present around the network of Tps. (**B**) A closer view of the Tp (arrow) network with podoms (curved arrow) forming a labyrinthine system within the interstitial tissue of the seminiferous tubules is shown. Large and small vesicles are also clearly visible near the Tps. Tc: telocyte; Bv: blood vessel; Cf: collagen fibres. The scale bars represent 10 μm (**A**) and 2 μm (**B**).

Furthermore, the TCs were frequently located around blood vessels. Complete TCs and their Tps near large blood vessels were examined (Fig. 7). The vasculature of the TCs included small cell bodies with extremely long, thin prolongations within the interstitial tissue, which contained small numbers of adjacent fibres, whereas the lumina of the blood vessels were clear (no fibres) with the exceptions of the red blood cells (Figs. 7 and 8A). We also observed that the prolongations of the vascular TCs exhibited greater numbers of bands and secreted various numbers of vesicles into the extracellular compartment (Fig. 8A and B). Furthermore, homocellular junctions, such as end‐to‐side membrane contacts (less than 13 nm) known as nanocontacts, were observed between the Tps surrounding the blood vessels (Fig. 9A and B). Telocytes were also detected around the large blood vessels and formed a labyrinthine system of overlapping Tps that was embedded in collagen fibres within the interstitial tissue of seminiferous tubules. Some uncommon junctions were also observed between the Tps and cell bodies of the TCs (Fig. 10A). Electron microscopy also revealed octopus‐like TCs with extremely twisted Tps embedded in the collagen fibres within the interstitial tissue (Fig. 10A). These Tps formed a labyrinthine system with three‐dimensional convolutions that overlapped each other and were also involved in the secretion of a large number of vesicles (Fig. 10B).

### IHC

**Figure 11:**
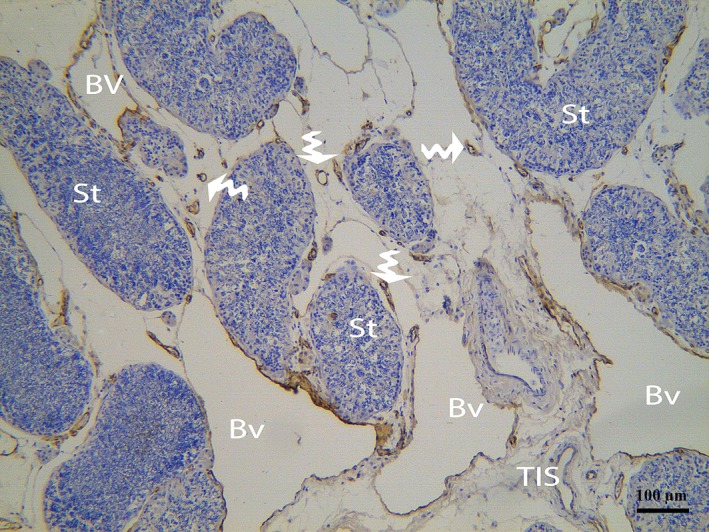
IHC photomicrograph of a turtle testis for CD34. CD34‐positive endothelial cells of several large and small blood vessels (curved arrow) are present between the seminiferous tubules. Bv: blood vessel; St: seminiferous tubules; TIS: testis interstitial tissue. The scale bars represents 100 μm.

**Figure 12:**
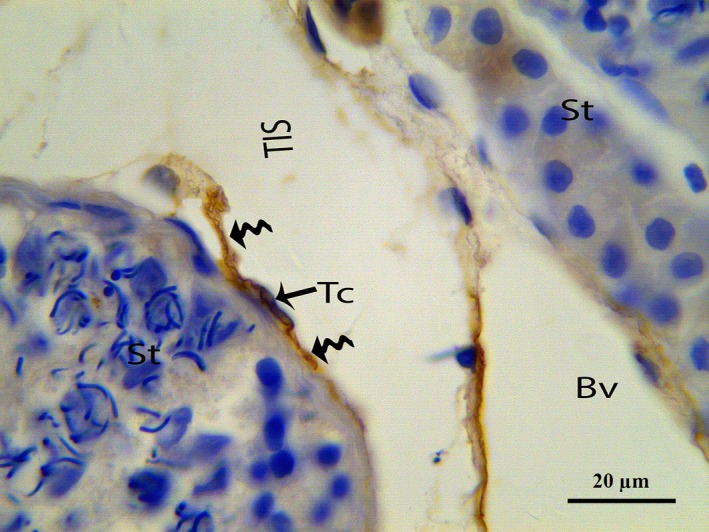
IHC photomicrograph of a turtle testis for CD34. CD34‐positive TCs with long thin Tps (curved arrow) extending from the cell body and bordering the seminiferous tubules are shown. Tc: telocyte; Tp: telopode; St: seminiferous tubules; TIS: testis interstitial tissue; Bv: blood vessel. The scale bars represents 20 μm.

**Figure 13:**
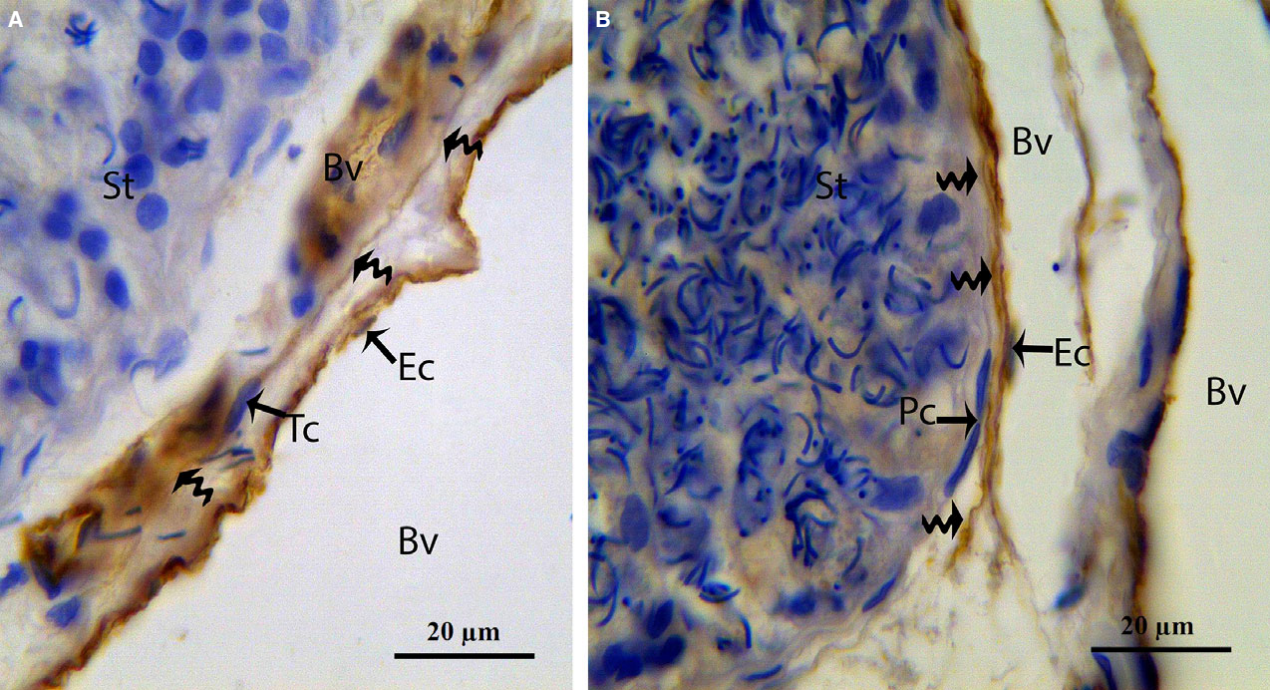
IHC photomicrograph of a turtle testis for CD34. (**A**) Spindle‐shaped CD34‐positive TCs with long thin prolongations (curved arrow) are present between blood vessels and seminiferous tubules. (**B**) TC with an extermly long Tp (curved arrow) form close contact with peritubular cells and blood vessel. Tc: telocyte; Tp: telopode; St: seminiferous tubules; Bv: blood vessel; Ec: endothelial cells. The scale bars represent 20 μm (**A** and **B**).

**Figure 14:**
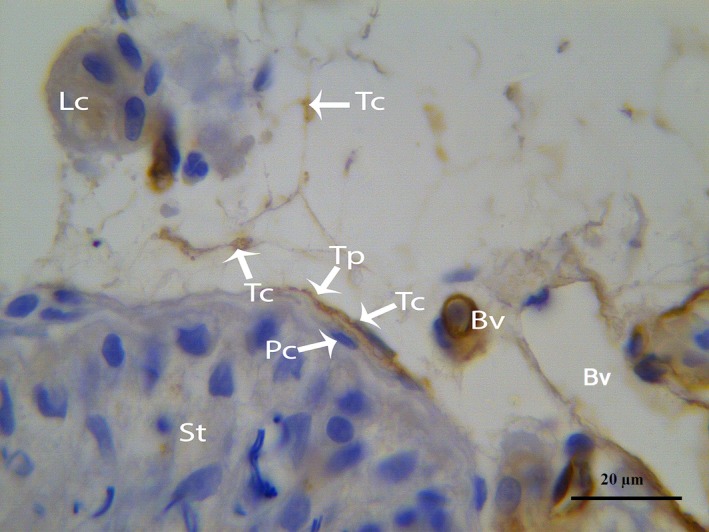
IHC photomicrograph of a turtle testis for CD34. Several CD34‐positive TCs with thin Tps are present in close proximity to peritubular and Leydig cells and blood vessels. Tc: telocye; Tp: telopode; St: seminiferous tubules; Pc: peritubular cell; Lc: Leydig cell. The scale bars represent 20 μm.

In our study, the endothelium and TCs were positively labelled for CD34, consistent with previous reports 14, 25, 29. The immunostaining revealed numerous positive endothelial cells in the small and large blood vessels in the connective tissue between the seminiferous tubules of the testes (Fig. 11). CD34 was expressed in the TC cell bodies and their cellular elongations, and the TCs were observed to be bipolar in shape with thin prolongations that extended to surround the adjacent Pc of the seminiferous tubules (Fig. 12). The CD34‐positive TCs were also located in the interstitial tissue between the seminiferous tubules and the endothelium of the blood vesselss (Fig. 13A). The extermly long Tp were present in the close contact with the Pc and blood vessel (Fig. 13B). Additionally, CD34‐positive TCs were found in the perivascular connective tissue, and these cells had three or more prolongations surrounding the nearby Leydig cells and blood vessels (Fig. 14). The shapes and distributions of the CD34‐positive TCs agreed with our TEM results.

## Discussion

In the past few years, numerous studies have demonstrated the existence of a novel type of cell, *i.e*., TCs, in different organs that had previously been termed ICLC. This study provides the first confirmation of the existence of TCs within the interstitial tissue (*i.e*., loose connective tissue) and the differentiation of these cells from the Pc of the testes of the soft‐shelled turtle based on the diagnostic criteria for TCs recommended by Popescu and Faussone‐Pellegrini 4. At present, only TEM allows of the definitive identification of TCs 4, 19. Through TEM, we discovered cells with morphologies similar to those of TCs present within the interstitial tissue of the testes of the soft‐shelled turtles. The cells identified in this study had similar morphologies and satisfied all of the previously established criteria for the identification of TCs and Tps. We found TCs with two to three Tps depending upon the site and the angle of the section. The Tps were tens to hundreds of micrometres in length as measured on the TEM images. However, 3D imaging of the TCs with FIB‐SEM technology revealed accurate reconstructions of the 3D volumes of the TCs and permitted imaging of areas that were several hundred serial sections in length 37. One of our primary hypotheses was that the morphology of Pc could lead to confusion with TCs, but our findings clearly indicated that the processes of the Pc had short, thick, spine‐like unidirectional outgrowths. Furthermore, the processes of the Pc contained abundant actin filaments with focal bodies, and the nuclei also exhibited irregularities (*i.e*., concertina nucleus‐like features) 26, 39. In contrast, the processes (Tps) of the TCs exhibited a different phenotype that was moniliform with alternating thin segments (podomers) and dilated bead‐like regions known as podoms 23, 27. Additionally, we also found that TCs were present in the outer layer surrounding the Pc within the interstitial tissue. It has been reported that Pc not only provide structural support to the seminiferous tubules but also play important roles in the blood‐testis barrier 40 and the transport of spermatozoa and testicular fluid due to their contractile nature 2; thus, it can be hypothesized that the TCs, as well as Pc, are involved in the abovementioned functions.

The present study revealed the presence of TCs that were directly connected to Leydig cells, which suggests that TCs are indirectly involved in the secretion of testosterone, rostenedione and dehydropiandrosterone 38. We reported that TCs and their Tps are located in close proximity to several large and small blood vessels. Some previous studies have demonstrated that the processes of TCs may act as cellular guides for immune cells *via* the blood stream 6 and that TCs are ‘strategically positioned’ in tissues between blood capillaries 4. Additionally, we also observed that the vascular TCs secreted more vesicles and bands in the Tps than the TCs that were located within other structures. The presence of a large number of vesicles appears to be a conserved feature of TCs regardless of their location. Here, this vesicle density seems to evidence cell‐to‐cell communication *via* the direct stimulation of target cells or receptor‐mediated interactions 41. However, it has also been reported that TCs can function as ‘hormonal sensors’ in the human reproductive tract because they express progesterone and oestrogen receptors 34, 35. Furthermore, a role of TCs in juxta‐ and/or paracrine signalling was previously been proposed 42, and it is now proven that TCs are capable of performing such a role. Telocytes establish hetero‐ and homocellular junctions 43, 44 and are capable of releasing extracellular vesicles 12, 14. Our results are in accordance with recent findings because we clearly observed homo‐ and heterocellular junctions and released extracellular vesicles. A previous study suggested that the interactions between SCs, Pc, Leydig cells, and germ cells are essential for the regulation of spermatogenesis 45, 46. The present study provided sufficient evidence of the existence of TCs in the testis, thus, we hypothesized that the TCs, as well as the above‐mentioned cells, played an important role in the regulation of spermatogenesis.

The present study observed that TCs are found embedded in the collagen fibres that may be involved in the remodelling, regeneration and repair of the interstitial tissue of the testis. Our findings are in line with those of previous studies that have reported that skin TCs are found in close proximity to collagen and elastic fibres 47, 48.

The identification of TCs in this study was also performed immunohistochemically using CD34 antibodies. However, the TCs and endothelial cells of the blood vessels exhibited immunoreactivity but were clearly distinguishable by their different morphologies. The TCs appeared as small pyriform or spindle‐shaped cells with two or more thin, long processes surrounded on both sides by a small amount of fibres 12. In contrast, the endothelial cells appeared flat and touched fibres on only one side due to the lumina of the blood vessels. These endothelial cells provide a physical interface between the blood and the surrounding tissue and regulate nutrient and blood component trafficking 49. In addition to that the location of our CD34 positive TCs are in line with the previous study 50, 51, which gives the evidence that CD34‐positive stromal cells were distributed in the outer layer of seminiferous tubules and between tubules or around Leydig cells in a reticular network in human testis, thus, the contact of the TCs may play an important role in the testes. In the turtle testes, both the morphologies and the locations of the labelled cells were entirely in line with our TEM findings. Telocytes may be a novel target cell type for the prevention and treatment of testicular disorders. Investigations of the mechanisms underlying the interaction between TCs and other cells and explorations of the potential biofunctions of TCs in some pathological conditions of the testes are still needed.

## Conflicts of interest

The authors confirm that there are no conflicts of interest.
